# Evidence of association between single-nucleotide polymorphisms in lipid metabolism-related genes and type 2 diabetes mellitus in a Chinese population

**DOI:** 10.7150/ijms.53004

**Published:** 2021-01-01

**Authors:** Yiping Li, Siqi He, Chuanyin Li, Keyu Shen, Man Yang, Wenyu Tao, Ying Yang, Li Shi, Yufeng Yao

**Affiliations:** 1 Department of Endocrinology and Metabolism, The Second People's Hospital of Yunnan Province & The Affiliated Hospital of Yunnan University, Kunming 650021, Yunnan, China.; 2 Dali University, Dali 671000, Yunnan, China.; 3 Institute of Medical Biology, Chinese Academy of Medical Sciences & Peking Union Medical College, Kunming 650118, Yunnan, China.; 4 Department of Medicine, Dentistry and Healthy Science, The University of Melbourne, Melbourne VIC3010, Australia.

**Keywords:** Polymorphisms, Chinese population, lipid metabolism-related genes, T2DM

## Abstract

**Background:** Type 2 diabetes mellitus (T2DM) is a complex chronic metabolic disorder triggered by insulin resistance in peripheral tissues. Evidence has shown that lipid metabolism and related genetic factors lead to insulin resistance. Hence, it is meaningful to investigate the association between single-nucleotide polymorphisms (SNPs) in lipid metabolism-related genes and T2DM.

**Methods:** A total of 1,194 subjects with T2DM and 1,274 Non-diabetic subjects (NDM) were enrolled. Five SNPs in three genes (rs864745 in *JAZF1*, rs35767 in *IGF1*, and rs4376068, rs4402960, and rs6769511 in *IGF2BP2*) that contribute to insulin resistance involving lipid metabolism were genotyped using the MassArray method in a Chinese population.

**Results:** The allele and genotypes of rs6769511 in *IGF2BP2* were associated with T2DM (P=0.009 and P=0.002, respectively). In inheritance model analysis, compared with the T/T-C/T genotype, the C/C genotype of rs6769511 in *IGF2BP2* was a risk factor for the development of T2DM (P<0.001, odds ratio [OR] =1.76; 95% confidence interval [CI]: 1.29-2.42). Haplotype analysis revealed associations of the rs4376068-rs4402960-rs6769511 haplotypes in *IGF2BP2* with the development of T2DM (P=0.015). Additionally, rs4376068C-rs4402960T-rs6769511C was a risk haplotype for T2DM (OR=1.179; 95% CI: 1.033-1.346).

**Conclusion:** The rs6769511 in *IGF2BP2* was associated with T2DM susceptibility, and the rs4376068-rs4402960-rs6769511 haplotypes in *IGF2BP2* was associated with the development of T2DM in a Chinese population.

## Introduction

Type 2 diabetes mellitus (T2DM) is a chronic multifactorial hyperglycaemic disease characterised by insulin resistance and impaired insulin secretion. Compared with insulin secretion, insulin resistance is the basis and major feature of T2DM 1 and is an inherited defect that initiates the diabetic condition in the majority of patients with T2DM 2. Accumulated data have revealed that lipid abnormalities are associated with insulin resistance and contribute to T2DM 3, 4. Studies have also revealed lipid metabolism-related genes and their single-nucleotide polymorphisms (SNPs) associated with insulin resistance and the development of T2DM 5-12.

Recent evidence has shown that juxtaposed with another zinc finger gene 1 (JAZF1), insulin-like growth factor 1 (IGF1), and insulin-like growth factor 2 mRNA-binding protein 2 (IGF2BP2), which are expressed in insulin-resistant tissue, are involved in lipid metabolism and are associated with insulin resistance 7, 8, 13-18. In adipocytes, JAZF1 inhibits the accumulation of fatty acids and triglycerides 15. In the liver, JAZF1 enhances fatty acid β-oxidation 16. IGF-1 downregulates free fatty acid and increases fatty acid β oxidation during fasting 9. Hepatic IGF2BP2 deficiency leads to a progressive deposition of hepatic triglycerides and elevated circulating triglycerides due to reduction of fatty acid oxidation, while global IGF2BP2 deficiency is accompanied by reduced circulating fatty acids and triglycerides 13, 14.

In addition to the indirect role of JAZF1, IGF1, and IGF2BP2 in insulin resistance involving lipid metabolism, the three proteins can also have direct effects on glucose metabolism 5, 16, 19. The genes encoding these proteins, as well as their SNPs, have been associated with T2DM and metabolic traits 6, 11, 20-26. For example, Long *et al*. reported that rs6769511C in *IGF2BP2* was associated with the risk of T2DM in African Americans 22. Then, Kuo *et al*. also observed that the rs6769511C allele was associated with T2DM in a Chinese Han population 26.

In the current study, 1,194 patients with T2DM and 1,274 Non-diabetic (NDM) subjects in a Chinese population were enrolled. Five SNPs in three genes (rs864745 in *JAZF1*, rs35767 in *IGF1*, and rs4376068, rs4402960, and rs6769511 in *IGF2BP2*) contributing to insulin resistance involving lipid metabolism were genotyped. We also investigated the association of the five SNPs and metabolic traits in the T2DM and NDM groups in the Chinese population.

## Materials and Methods

### Ethics statement

This study was approved by the Institutional Review Board of the Second People's Hospital of Yunnan Province prior to beginning the study. The study protocols complied with the principles expressed in the Helsinki Declaration of 1975, which was revised in 2008. Written informed consent was obtained from each participant.

### Subjects

A total of 1,194 patients (762 men and 432 women) diagnosed with T2DM at the Second People's Hospital of Yunnan Province from February 2017 to July 2019 were enrolled. The diagnostic standard of T2DM conformed to the World Health Organization criteria published in 1999 and the American Diabetes Association guidelines of 2020 27. Insulin secretion and C-peptide levels were measured to exclude subjects with type 1 diabetes. For subjects <40 years old, islet cell autoantibodies and glutamic acid decarboxylase autoantibodies were detected to exclude autoantibody positive subjects. Gestational women were not included. Specific types of diabetes (e.g., glucocorticoid-induced diabetes) were excluded based on medical history. The NDM group included 1,274 subjects (782 men and 492 women) with no family history of diabetes mellitus. These subjects were recruited from among individuals undergoing routine health check-ups at the Second People's Hospital of Yunnan Province. Subjects with impaired glucose tolerance and/or glycosylated haemoglobin (HbA1C) levels ≥ 5.7% were excluded from the NDM group 27. Subjects with hypertension or coronary heart disease were also excluded. All participants described themselves as being of Han ethnicity.

### Laboratory measurements

Venous blood samples were collected the morning after the subjects had fasted for 12 hours. Fasting plasma glucose (FPG), total cholesterol (TC), triglycerides (TG), high-density lipoprotein cholesterol (HDL-C), low-density lipoprotein cholesterol (LDL-C), and HbA1C were measured. All laboratory measurements were performed using a 7600-020 automatic analyser (Hitachi, Tokyo, Japan).

### Selection and genotyping of SNPs

In the current study, we focused on three genes (*JAZF1*, *IGF1,* and* IGF2BP2*) involved in lipid metabolism, which have been shown to affect insulin resistance 5-11, 13-18. The SNP rs864745 in *JAZF1* is located in intron 1 28, 29 and rs35767 is located in the *IGF1 promoter* 30. The three SNPs (rs4376068, rs4402960, and rs6769511) of *IGF2BP2* are all located in intron 2 6, 31.

The QIAamp Blood Mini Kit (Qiagen, Hilden, Germany) was used to extract genomic DNA from peripheral lymphocytes. The aforementioned five SNPs in the three genes were genotyped using the MassArray Analyser 4.0 (Agena Bioscience, Inc., San Diego, CA, USA) as previously described 32. Matrix-assisted laser desorption/ionization time-of-flight mass spectrometry (MALDI-TOF MS) was performed using a device from Agena Bioscience. SNP genotyping was performed using a SpectroCHIP array, and the raw genotyping data were obtained using TYPER4.0 software (Agena Bioscience).

### Statistical analyses

Age, glucose, and lipid metabolic parameters (TC, HDL-C, TG, LDL-C, FPG, and HbA1C) were compared between the T2DM and NDM groups using Student's t test. Sex in the T2DM and NDM groups was compared using the Chi-square test. All polymorphic loci were tested for deviation from the Hardy-Weinberg equilibrium in the NDM group with a threshold of 0.05. Risks were estimated by odds ratios (OR) with 95% confidence intervals (95% CI). Linkage disequilibrium (LD) among these SNPs was estimated. The LD coefficient D was calculated using SHEsis software 33, 34. The distribution and differences in the haplotypes between the T2DM and NDM groups were determined using SHEsis software. Haplotype frequencies <0.03 were ignored in the analysis 33, 34. The association between each SNP and T2DM was analysed for mode of inheritance using SNPStats 35. Power-analysis was performed using power and sample size calculations 36. Bonferroni correction for multiple comparisons was used for each SNP and haplotype.

## Results

### Subject characteristics

Table 1 lists the clinical characteristics and glucose and lipid metabolic parameters of the enrolled subjects. There were no differences in age or sex between the T2DM and NDM groups, or in age between the male and female subjects in both groups (all P>0.05). However, significant differences were evident in the glucose and lipid metabolic parameters (TC, TG, HDL-C, LDL-C, FPG, and HbA1C) between the T2DM and NDM groups (all P<0.001; Table 1). T2DM subjects displayed dyslipidaemia and dysglycemia with higher TC, TG, LDL-C, FPG, and HbA1C and lower HDL-C, compared with NDM subjects.

### Association of the five SNPs with T2DM

The allele and genotype frequencies for the five SNPs in the candidate T2DM susceptible genes are listed in Table 2. The genotype frequencies for the five SNPs in the NDM group were in Hardy-Weinberg equilibrium (P>0.05; Table 2). The genotype frequency distribution of rs4376068 and rs4402960 in *IGF2BP2* showed a different trend between the NDM and T2DM groups (P=0.051 and P=0.020, respectively). The allele frequency distribution of rs4376068 and rs4402960 in *IGF2BP2* showed a different trend between the NDM and T2DM groups (P=0.022 and P=0.011, respectively). However, after Bonferroni correction, the alleles and genotypes of these two SNPs showed no significant difference between the NDM and T2DM groups. For the rs6769511 SNP in *IGF2BP2*, alleles and genotypes showed significant differences between the T2DM and NDM groups after Bonferroni correction (P=0.009 and P=0.002, respectively). The C allele of rs6769511 was a risk factor for the development of T2DM (OR=1.185; 95% CI: 1.043-1.347). The allele and genotype frequency distribution of the rs864745 in *JAZF1* and rs35767 in* IGF1* failed to show an association with T2DM (P>0.05).

### Model of inheritance analysis

Data on the inheritance models (codominant, dominant, recessive, overdominant, and log-additive) of these SNPs are listed in Tables 3 to 7. The lowest Akaike information criterion (AIC) and Bayesian information criterion (BIC) were used to determine the best fit model for each SNP 35. For rs6769511 in *IGF2BP2*, the best-fit inheritance model with the lowest AIC and BIC was recessive. Compared with the T/T-C/T genotype, the C/C genotype was a risk factor for T2DM (P<0.001, OR=1.76; 95% CI: 1.29-2.42). In addition, the best-fit inheritance model with the lowest AIC and BIC for rs4376068 and rs4402960 in *IGF2BP2* was log-additive. However, both rs4376068 and rs4402960 failed to show significant differences between the T2DM and NDM groups after Bonferroni correction (P=0.020 and P=0.010, respectively). The other SNPs did not show any association with T2DM (P>0.05).

### LD among the three SNPs in *IGF2BP2*

LD is displayed as a pairwise D'. The D' values were defined in the range (-1, 1), with a value of 1 representing perfect disequilibrium. A D' value >0.8 indicated the existence of different loci in the LD. Based on the location of the rs4376068, rs4402960, and rs6769511 SNPs in chromosome 3, the LD among these SNPs was estimated. The three SNPs were in LD (D′>0.9). D′ values obtained from all individuals were 0.969 between rs4376068 and rs4402960, 0.936 between rs4376068 and rs6769511, and 0.930 between rs4402960 and rs6769511.

### Association of haplotypes of SNPs with T2DM

According to the D′ value (D′>0.8), the haplotypes of the three SNPs (rs4376068-rs4402960-rs6769511) in *IGF2BP2* were constructed. The frequency of haplotype rs4376068C-rs4402960T-rs6769511C was 0.248 in T2DM and 0.221 in NDM patients. The frequency of haplotype rs4376068A-rs4402960G-rs6769511T was 0.708 in T2DM and 0.743 in NDM patients. The haplotypes showed significant differences between the T2DM and NDM groups after Bonferroni correction (P=0.015; Table 8). rs4376068C-rs4402960T-rs6769511C was implicated as a high-risk haplotype of T2DM (OR=1.179, 95%CI: 1.033-1.346), while rs4376068A-rs4402960G-rs6769511T could be a protective haplotype against T2DM (OR=0.848, 95% CI: 0.743-0.969).

## Discussion

We investigated the influence of 5 SNPs in 3 genes (rs864745 in *JAZF1*, rs35767 in *IGF1*, and rs4376068, rs4402960, and rs6769511 in *IGF2BP2*) on T2DM in a Chinese population. These SNPs contribute to insulin resistance by affecting lipid metabolism in T2DM.

*IGF2BP2* has a comprehensive role in lipid metabolism 13, 14. Dai *et al*. reported that *IGF2BP2* null mice are hypolipidemic, glucose tolerant, and insulin sensitive 13. The loss of *IGF2BP2* results in an increase in uncoupling protein-1 (UCP1), which contributes to resistance to obesity and a beneficial metabolic profile 13. Long *et al*. reported the association of the rs6769511C allele in *IGF2BP2* with the risk of T2DM in African Americans 22. Kuo *et al*. also observed that the rs6769511C allele was associated with T2DM in a Chinese Han population 26. Haljas *et al*. identified an association between the rs6769511C allele and T2DM in a meta-analysis 37. Our current results also indicate an association between the allele and genotypes of rs6769511 with T2DM. The C allele of rs6769511 was implicated as a risk factor for the development of T2DM (P=0.009, OR=1.185; 95% CI: 1.043-1.347). Horikawa *et al*. and Omori *et al*. reported the association of rs4402960T with the risk of developing T2DM in Japanese populations 20, 21. Wu *et al*. and Huang *et al*. also found that the rs4402960T allele plays an important role in the predisposition of T2DM in the Chinese population 38, 39. Several subsequent studies reported the association of the rs4402960T allele with the risk of T2DM in different populations 31, 40-42. One of the reasons for the association of rs4402960T with T2DM could be the association of the T allele of rs4402960 with insulin sensitivity, which affects all glucose tolerance phenotypes, or the significantly increased insulin resistance of genotype TT, determined by homoeostasis model assessment for insulin resistance (HOMA-IR) and increased levels of *IGF2BP2* mRNA in adipocytes 5, 6. Therefore, rs4402960T could lead to disorders of T2DM related metabolic traits by increasing *IGF2BP2* mRNA levels. In the current study, we also found that the T allele of rs4402960 could be a risk for T2DM (P=0.011, OR=1.181; 95% CI: 1.039-1.342). However, after Bonferroni correction, the SNP showed no significant difference between T2DM and NDM. One of the reasons for the discrepancies between the current and prior results could be the sample size. Our sample size was relatively moderate. We calculated the statistical power of rs4402960 using PS software 36 and it reached 0.487. If our sample size was greater, such as 2000 cases and 2000 controls, the statistical power would reach 0.746. Thus, future studies should include more subjects to conclusively identify the association between these SNPs and T2DM. Moreover, in the current study, we observed that the C allele of rs4376068 could be a risk for T2DM (P=0.022). There was no significant difference between T2DM and NDM after Bonferroni correction. However, since no other studies have addressed the association between rs4376068 and T2DM, our results must be confirmed in other studies.

In the current study, the haplotypes rs4376068C-rs4402960T-rs6769511C and rs4376068A-rs4402960G-rs6769511T in *IGF2BP2* acted as risk and protective haplotypes in T2DM, respectively. Interestingly, the haplotypes that included the risk or protective alleles of the three SNPs were also risk and protective haplotypes in T2DM. These findings indicate that the three SNPs in the polymorphic genes may have combinatorial effects on T2DM susceptibility. In addition, compared with the effect of a single locus, the role of haplotypes on complex T2DM may be more remarkable. The reason for the positive haplotype association with T2DM and not the single locus could be that the single locus has a weak effect on the disease, while the linkage of loci has a reinforcing effect. Christodoulou and Avgeris *et al*. recently investigated the expression patterns of T2DM susceptibility genes and determined their individual transcript variants (tv) in peripheral blood of T2DM patients and controls (CT_RF-_ group: no risk factors for the development of T2D; CT_RF+_ group: risk factors for the disease). Their data showed that the levels of tv7 of *IGF2BP2* (which lacks exons 1 and 2 compared to the canonical tv1) exhibited a significant stepwise upregulation from CT_RF-_ to CT_RF+_ in T2DM patients. Since rs4376068, rs4402960, and rs6769511 were all located in intron 2 of *IGF2BP2*, these SNPs could influence the transcript variants of *IGF2BP2*, which was associated with the development of T2DM 43.

*JAZF1* serves as a metabolic regulator with the function of improving lipid metabolism and resisting hyperglycaemia through multiple metabolic signalling pathways in T2DM 16. The favourable effects of *JAZF1* on lipid metabolism have been observed in adipocytes and hepatocytes 15, 17, 18. In addition to its role in improving lipid metabolism, *JAZF1* has an important role in promoting glucose transport and enhancing insulin sensitivity in both liver and adipose tissue 7. Several studies have reported an association between the T allele and TT genotype of rs864745 with the development of T2DM in African American, ethnic Chinese She, Japanese, and Uyghur populations 12, 23, 24, 29, 44. However, Deng *et al*. did not find an association between rs864745 and T2DM risk in a Chinese Han population 24, which is consistent with the current results. Similarly, Gamboa-Meléndez *et al*. found no association between rs864745 and T2DM in a Mexican Mestizo population (P>0.05) 45. One of the reasons for the study differences could be the differences in ethnic and genetic backgrounds.

*IGF1* is a key factor supporting growth and regulating metabolism. Nearly 30 years ago, Schalch *et al*. reported that *IGF-1* was associated with significant reductions in fasting blood glucose and serum insulin 19. More recently, Dupuis *et al*. documented that the association of the rs35767G allele in *IGF1* was associated with HOMA-IR and T2DM in a European population 10. Dotta *et al*. reported that the rs35767GG genotype exhibited circulating concentrations of *IGF1* and lower insulin sensitivity in Europeans 11. However, Zhang *et al*. found that the A allele of rs35767 contributed to the risk of developing T2DM in a Chinese Han population 25. Presently, we did not find evidence of an association between rs35767 and T2DM. One of the reasons for the discrepancies in the risk allele of rs35767 between European and Chinese populations could be the genetic background. The frequency of rs35767A was approximately 25% to 37% in the Asian population, but only 16% in the European population (https://asia.ensembl.org/Homo_sapiens/Variation/Population?db=core;r=12:102481291-102482291;v=rs35767;vdb=variation;vf=95676582). The discrepancy between our data and that of Zhang *et al*. could be due to the different sample sizes. A total of 1,194 cases and 1,274 controls were enrolled in the current study. Zhang *et al*. enrolled 244 cases and 142 controls. Genetic differences could be another reason for this. The samples in the current study were from Yunnan Province, southwest China. The sample in the study by Zhang *et al*. was from Hebei province in the north of China. Several studies have reported that the genetic background is distinct between northern and southern Han, as identified using short tandem repeats, SNP, and HLA genes 46-49. Moreover, the risks for the same disease in the Han Chinese population involving certain SNPs could be different between the northern and southern Han.

One limitation of the current study is that a relatively moderate sample size may limit its statistical power; the statistical power for the effect of the rs6769511 SNP in IGF2BP2 was 0.512, which was relatively lower. Thus, a larger population should be investigated, and the results of the current study validated in future studies.

## Conclusions

In the current study, we focused on SNPs of three genes (*JAZF1*, *IGF1*, and *IGF2BP2*) which have been shown to affect lipid metabolism and insulin resistance. Our results revealed a significant association between rs6769511 in *IGF2BP2* with T2DM, indicating that the SNP could be related to insulin resistance involving lipid metabolism. Moreover, the rs4376068-rs4402960-rs6769511 haplotypes in *IGF2BP2* was associated with the development of T2DM. The results indicated that the functional effects of these polymorphisms associated with lipid metabolism and insulin resistance need to be investigated further.

## Figures and Tables

**Table 1:** Clinical characteristics and glucose and lipid metabolic parameters of the subjects enrolled in the present study

	Type 2 diabetes (T2DM)	Non-diabetic subject (NDM)	*P* value
N	1,194	1,274	
Age, y	52.490±12.101	52.764±10.543	0.550
Sex (M/F)	762/432	782/492	0.211
Age/M, y	51.366±12.399	51.715±10.557	0.552
Age/F, y	54.472±11.300	54.431±10.315	0.954
Total cholesterol, mmol/L	4.816±1.076	4.516±1.053	<0.001
Triglycerides, mmol/L	2.459±2.120	1.749±1.277	<0.001
High-density lipoprotein-cholesterol, mmol/L	1.093±0.281	1.291±0.424	<0.001
Low-density lipoprotein-cholesterol, mmol/L	2.794±0.969	2.632±0.869	<0.001
Fasting plasma glucose, mmol/L	7.895±2.503	5.006±0.527	<0.001
HbA1C (%)	8.990±2.817	5.138±0.356	<0.001

**Table 2:** Comparison of genotypic and allelic distribution of five SNPs (rs864745, rs35767, rs4376068, rs4402960 and rs6769511) between the Type 2 diabetes (T2DM) and Non-diabetic subject (NDM)

SNPs	Allele		X²	*P* value	Odds ratio (95% CI)	Genotypes			X²	*P* value	H-W
rs864745	T (freq)	C (freq)				T/T (freq)	T/C (freq)	C/C (freq)			
T2DM	1869 (0.783)	519 (0.217)	1.353	0.245	1.083 (0.947-1.238)	736 (0.616)	397 (0.332)	61 (0.051)	3.796	0.150	0.434
NDM	1959 (0.769)	589 (0.231)				744 (0.584)	471 (0.370)	59 (0.046)			0.152
rs35767	G (freq)	A (freq)				G/G (freq)	G/A (freq)	A/A (freq)			
T2DM	1538 (0.644)	850 (0.356)	1.590	0.207	1.077 (0.959-1.210)	513 (0.430)	512 (0.429)	169 (0.142)	4.278	0.118	0.025
NDM	1597 (0.627)	951 (0.373)				500 (0.392)	597 (0.469)	177 (0.139)			0.955
rs4376068	C(freq)	A (freq)				A/A (freq)	C/A (freq)	C/C (freq)			
T2DM	627 (0.263)	1761 (0.737)	5.280	0.022	1.164 (1.022-1.324)	649 (0.544)	463 (0.388)	82 (0.069)	5.933	0.051	0.963
NDM	597 (0.234)	1951 (0.766)				740 (0.581)	471 (0.370)	63 (0.049)			0.279
rs4402960	T (freq)	G (freq)				G/G (freq)	T/G (freq)	T/T (freq)			
T2DM	645 (0.270)	1743 (0.730)	6.450	0.011	1.181 (1.039-1.342)	635 (0.532)	473 (0.396)	86 (0.072)	7.779	0.020	0.871
NDM	608 (0.239)	1940 (0.761)				728 (0.571)	484 (0.380)	62 (0.049)			0.104
rs6769511	C (freq)	T (freq)				C/C (freq)	C/T (freq)	T/T (freq)			
T2DM	655 (0.274)	1733 (0.726)	6.823	0.009	1.185 (1.043-1.347)	107 (0.090)	441 (0.369)	646 (0.541)	12.989	0.002	0.013
NDM	616 (0.242)	1932 (0.758)				67 (0.053)	482 (0.378)	725 (0.569)			0.254

Note: Significant threshold after Bonferroni correction for multiple comparisons is less than 0.010 (0.05/5); H-W: Hardy-Weinberg equilibrium; CI: confidence interval.

**Table 3:** Different inheritance models analysis of the SNP rs6769511 between the Type 2 diabetes (T2DM) and Non-diabetic subject (NDM)

Model	Genotype	NDM	T2DM	Odds ratio (95% CI)	*P* value	AIC	BIC
Codominant	T/T	725 (56.9%)	646 (54.1%)	1.00	0.002	3414.2	3443.3
	C/T	482 (37.8%)	441 (36.9%)	1.03(0.87-1.22)			
	C/C	67 (5.3%)	107 (9%)	1.78 (1.29-2.47)			
Dominant	T/T	725 (56.9%)	646 (54.1%)	1.00	0.160	3423	3446.3
	C/T-C/C	549 (43.1%)	548 (45.9%)	1.12 (0.96-1.31)			
Recessive	T/T-C/T	1207 (94.7%)	1087 (91%)	1.00	<0.001	3412.3	3435.6
	C/C	67 (5.3%)	107 (9%)	1.76 (1.29-2.42)			
Overdominant	T/T-C/C	792 (62.2%)	753 (63.1%)	1.00	0.670	3424.8	3448.1
	C/T	482 (37.8%)	441 (36.9%)	0.96 (0.82-1.14)			
Log-additive	---	---	---	1.18 (1.04-1.34)	0.010	3418.4	3441.6

Note: Significant threshold after Bonferroni correction for multiple comparisons is less than 0.010 (0.05/5); AIC: Akaike information criterion; BIC: Bayesian information criterion; CI: confidence interval.

**Table 4:** Different inheritance models analysis of the SNP rs4402960 between the Type 2 diabetes (T2DM) and Non-diabetic subject (NDM)

Model	Genotype	NDM	T2DM	Odds ratio (95% CI)	*P* value	AIC	BIC
Codominant	G/G	728 (57.1%)	635 (53.2%)	1.00	0.021	3419.3	3448.4
	G/T	484 (38%)	473 (39.6%)	1.12 (0.95-1.33)			
	T/T	62 (4.9%)	86 (7.2%)	1.58 (1.12-2.23)			
Dominant	G/G	728 (57.1%)	635 (53.2%)	1.00	0.046	3421.0	3444.3
	G/T-T/T	546 (42.9%)	559 (46.8%)	1.18 (1.00-1.38)			
Recessive	G/G-G/T	1212 (95.1%)	1108 (92.8%)	1.00	0.016	3419.2	3442.4
	T/T	62 (4.9%)	86 (7.2%)	1.51 (1.08-2.11)			
Overdominant	G/G-T/T	790 (62%)	721 (60.4%)	1.00	0.390	3424.3	3447.5
	G/T	484 (38%)	473 (39.6%)	1.07 (0.91-1.26)			
Log-additive	---	---	---	1.19 (1.04-1.35)	0.010	3418.4	3441.6

Note: Significant threshold after Bonferroni correction for multiple comparisons is less than 0.010 (0.05/5); AIC: Akaike information criterion; BIC: Bayesian information criterion; CI: confidence interval.

**Table 5:** Different inheritance models analysis of the SNP rs4376068 between the Type 2 diabetes (T2DM) and Non-diabetic subject (NDM)

Model	Genotype	NDM	T2DM	Odds ratio (95% CI)	*P* value	AIC	BIC
Codominant	A/A	740 (58.1%)	649 (54.4%)	1.00	0.052	3421.1	3450.2
	A/C	471 (37%)	463 (38.8%)	1.12 (0.95-1.33)			
	C/C	63 (5%)	82 (6.9%)	1.48 (1.05-2.09)			
Dominant	A/A	740 (58.1%)	649 (54.4%)	1.00	0.059	3421.5	3444.7
	A/C-C/C	534 (41.9%)	545 (45.6%)	1.17 (0.99-1.37)			
Recessive	A/A-A/C	1211 (95%)	1112 (93.1%)	1.00	0.045	3421.0	3444.2
	C/C	63 (5%)	82 (6.9%)	1.41 (1.01-1.98)			
Overdominant	A/A-C/C	803 (63%)	731 (61.2%)	1.00	0.340	3424.1	3447.3
	A/C	471 (37%)	463 (38.8%)	1.08 (0.92-1.27)			
Log-additive	---	---	---	1.17 (1.02-1.33)	0.020	3419.6	3442.9

Note: Significant threshold after Bonferroni correction for multiple comparisons is less than 0.010 (0.05/5); AIC: Akaike information criterion; BIC: Bayesian information criterion; CI: confidence interval.

**Table 6:** Different inheritance models analysis of the SNPrs864745 between the Type 2 diabetes (T2DM) and Non-diabetic subject (NDM)

Model	Genotype	NDM	T2DM	Odds ratio (95% CI)	*P* value	AIC	BIC
Codominant	T/T	744 (58.4%)	736 (61.6%)	1.00	0.140	3423.1	3452.1
	C/T	471 (37%)	397 (33.2%)	0.85 (0.72-1.01)			
	C/C	59 (4.6%)	61 (5.1%)	1.05 (0.72-1.52)			
Dominant	T/T	744 (58.4%)	736 (61.6%)	1.00	0.096	3422.3	3445.5
	C/T-C/C	530 (41.6%)	458 (38.4%)	0.87 (0.74-1.02)			
Recessive	T/T-C/T	1215 (95.4%)	1133 (94.9%)	1.00	0.560	3424.7	3447.9
	C/C	59 (4.6%)	61 (5.1%)	1.11 (0.77-1.61)			
Overdominant	T/T-C/C	803 (63%)	797 (66.8%)	1.00	0.049	3421.1	3444.4
	C/T	471 (37%)	397 (33.2%)	0.85 (0.72-1.00)			
Log-additive	---	---	---	0.92 (0.81-1.06)	0.240	3423.6	3446.9

Note: Significant threshold after Bonferroni correction for multiple comparisons is less than 0.010 (0.05/5); AIC: Akaike information criterion; BIC: Bayesian information criterion; CI: confidence interval.

**Table 7:** Different inheritance models analysis of the SNP rs35767 between the Type 2 diabetes (T2DM) and Non-diabetic subject (NDM)

Model	Genotype	NDM	T2DM	Odds ratio (95% CI)	*P* value	AIC	BIC
Codominant	G/G	500 (39.2%)	513 (43%)	1.00	0.110	3422.7	3451.7
	G/A	597 (46.9%)	512 (42.9%)	0.84 (0.70-0.99)			
	A/A	177 (13.9%)	169 (14.2%)	0.93 (0.73-1.19)			
Dominant	G/G	500 (39.2%)	513 (43%)	1.00	0.061	3421.5	3444.8
	G/A-A/A	774 (60.8%)	681 (57%)	0.86 (0.73-1.01)			
Recessive	G/G-G/A	1097 (86.1%)	1025 (85.8%)	1.00	0.820	3425.0	3448.2
	A/A	177 (13.9%)	169 (14.2%)	1.03(0.82-1.29)			
Overdominant	G/G-A/A	677 (53.1%)	682 (57.1%)	1.00	0.044	3421.0	3444.2
	G/A	597 (46.9%)	512 (42.9%)	0.85 (0.72-1.00)			
Log-additive	---	---	---	0.93 (0.83-1.04)	0.220	3423.5	3446.8

Note: Significant threshold after Bonferroni correction for multiple comparisons is less than 0.010 (0.05/5); AIC: Akaike information criterion; BIC: Bayesian information criterion; CI: confidence interval.

**Table 8:** The haplotype analysis for SNPs located in intron 2 of the IGF2BP2 gene between the Type 2 diabetes (T2DM) and Non-diabetic subject (NDM)

Haplotypes (rs4376068-rs4402960-rs6769511)	T2DM (frequency)	NDM (frequency)	Chi^2^	*P* value	Odds ratio (95% CI)
CTC	592.48(0.248)	562.60(0.221)	5.923	0.015	1.179 [1.033~1.346]
AGT	1690.21(0.708)	1892.36(0.743)	5.923	0.015	0.848 [0.743~0.969]

Note: Significant threshold after Bonferroni correction for multiple comparisons is less than 0.025 (0.05/2); CI: confidence interval.
